# Satellite cell contribution to disease pathology in Duchenne muscular dystrophy

**DOI:** 10.3389/fphys.2023.1180980

**Published:** 2023-05-30

**Authors:** Kasun Kodippili, Michael A. Rudnicki

**Affiliations:** ^1^ The Sprott Centre for Stem Cell Research, Regenerative Medicine Program, Ottawa Hospital Research Institute, Ottawa, ON, Canada; ^2^ Department of Cellular and Molecular Medicine, Faculty of Medicine, University of Ottawa, Ottawa, ON, Canada

**Keywords:** Duchenne muscular dystrophy, satellite cells, skeletal muscle stem cells, muscle regeneration, myogenesis, dystrophin, asymmetric cell division, symmetric cell division

## Abstract

Progressive muscle weakness and degeneration characterize Duchenne muscular dystrophy (DMD), a lethal, x-linked neuromuscular disorder that affects 1 in 5,000 boys. Loss of dystrophin protein leads to recurrent muscle degeneration, progressive fibrosis, chronic inflammation, and dysfunction of skeletal muscle resident stem cells, called satellite cells. Unfortunately, there is currently no cure for DMD. In this mini review, we discuss how satellite cells in dystrophic muscle are functionally impaired, and how this contributes to the DMD pathology, and the tremendous potential of restoring endogenous satellite cell function as a viable treatment strategy to treat this debilitating and fatal disease.

## Introduction

Duchenne muscular dystrophy (DMD) is a rare yet well-characterized, severe and progressive muscle wasting disease. It is the most common type of muscular dystrophy with a global incidence of 1 in 5,000 live male births (Mendell et al., 2012; Mah et al., 2014; Ryder et al., 2017). Patients first present with symptoms such as difficulty climbing stairs and rising from a sitting or lying position, frequent falls, waddling gate, trouble running and jumping, etc., around 2–3 years of age. The progressive nature of this degenerative disease means that patients are often wheel-chair bound by the age of 10–12, and require assisted ventilation by the third decade of their life. Despite improvements in patient care and treatment strategies in the last few decades, most DMD patients die between 20–40 years of age from respiratory and/or cardiac complications (Mercuri et al., 2019).

## Etiology

DMD is a genetic disease caused by mutations in the dystrophin-encoding *DMD* gene on the X chromosome. Consequently, DMD is presented primarily in males, and is maternally inherited. With 79 exons, and spanning 2.2 Mb, the *DMD* gene is the largest known human gene (Koenig et al., 1987). The gene also carries a relatively high mutation rate, with about a third of all DMD cases caused by *de novo* germline mutations. Approximately one-third of all mutations in DMD patients are caused by deletions of one or more exons. Another 5%–15% are duplications, and about 20% are small mutations such as point mutations, deletions, or insertions (Aartsma-Rus et al., 2006). While these large deletions or duplications can occur anywhere in the gene, two mutation hotspots have been identified between exons 45–55 and 2–10 (Duan et al., 2021). In general, if mutations do not disrupt the open reading frame, a partially functional dystrophin protein, albeit shorter or longer in the middle, will be produced and result in a milder disease called Becker muscular dystrophy (BMD) (Koenig et al., 1989; Duan et al., 2021).

## Mechanisms and pathophysiology

**FIGURE 1:**
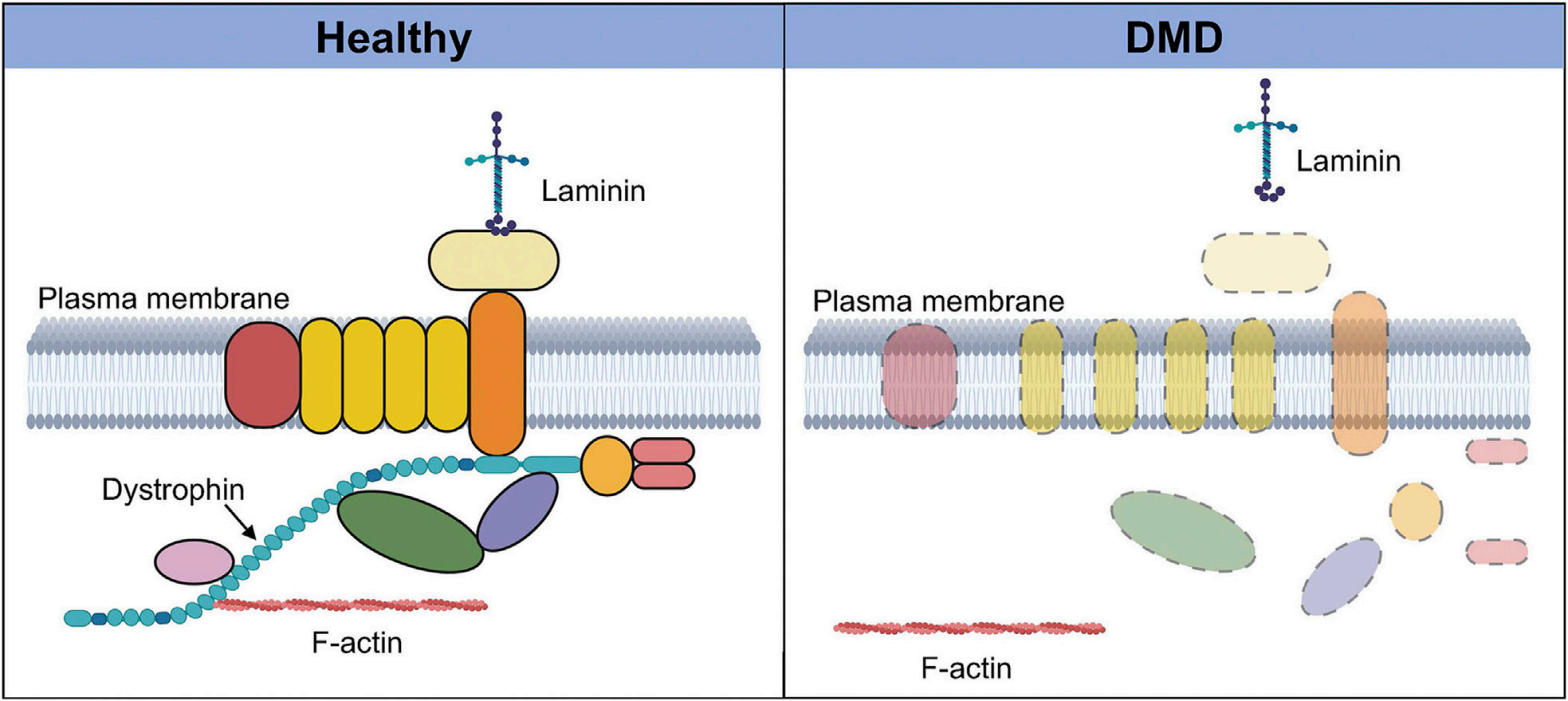
Dystrophin-associated protein complex in healthy and Duchenne muscular dystrophy (DMD) muscle. In healthy muscle (left), dystrophin and its binding partners together form a protein complex called the dystrophin-associated protein complex, which plays numerous signaling and structural roles. This highly organized transmembrane protein complex links the intracellular cytoskeleton with the sarcolemma and the extra-cellular matrix via laminin. In the absence of dystrophin in DMD muscle (right), this protein complex is disassembled, and there is delocalization and loss of expression of several components of the complex. The interaction between F-actin and the extracellular matrix is also lost, resulting in a wide range of deleterious effects that severely compromise the integrity and function of the tissue. Created with BioRender.com.

DMD results in the loss of the muscle isoform of dystrophin (Dp427m) (Hoffman et al., 1987). The dystrophin protein plays a key structural role in muscle where it links the internal cytoskeleton to the extracellular matrix and the dystrophin-associated protein complex (DAPC) (Ervasti and Campbell, 1993; Ervasti, 2007). The loss of dystrophin protein results in the disassembly of the DAPC, reduced expression levels of certain DAPC components, and the loss of the interaction between F-actin in cytoskeleton and the extracellular matrix (Figure 1). This, together with the loss of crucial signaling roles played by DAPC member proteins, leads to wide-ranging consequences including loss of myofiber integrity, membrane leakage, impaired muscle fiber contractile activity, and progressive muscle degeneration (Nowak and Davies, 2004; Guiraud et al., 2015).

## Muscle degeneration

Dystrophin, along with the DAPC, function as a molecular scaffold serving a mechanical function during muscle contraction. In healthy muscle, this linkage between the cytoskeleton, sarcolemma and extracellular matrix is crucial for weathering the force output and significant mechanical stress arising from each bout of muscle contraction. Even so, repetitive contractions can cause sarcolemmal defects and tears in healthy muscle as well, leading to muscle degeneration and inflammation (Ervasti and Sonnemann, 2008; Abdel-Salam et al., 2009). In response, tissue resident skeletal muscle stem cells (MuSCs, also called satellite cells) are activated and mobilized to repair the damage and replace lost muscle fibers (Dumont et al., 2015a). In dystrophic muscle, the sarcolemma is further susceptible to contraction-induced damage, and this increased sarcolemmal permeability results in the influx of calcium and other small molecules leading to cell dysfunction and death. Continued cell death ultimately leads to an imbalance in muscle degeneration and regeneration, and the deposition of fibrotic tissue in place of functional muscle, further exacerbating disease pathology. Unfortunately for DMD patients, lack of dystrophin protein has direct consequences on the function of satellite cells as well (Dumont and Rudnicki, 2016). This stem cell phenotype and its contribution to disease in DMD, along with the remarkable potential of stem cell-based treatments to alter the rates of muscle degeneration and regeneration, and disease progression will be the focus of this mini review.

## Satellite cells

Adult skeletal muscle, which accounts for about 30%–40% of the adult human body weight, is stable under homeostatic conditions, with only sporadic proliferation and fusion of satellite cells to compensate for muscle turnover from normal wear and tear. Yet, upon tissue injury, skeletal muscle demonstrates an incredible ability to regenerate. This is enabled by satellite cells, the tissue resident stem cell population. Satellite cells, independently discovered in 1961 by Alexander Mauro and Bernard Katz, are cells “wedged” between the basal lamina and plasma membrane of the muscle fiber (Mauro, 1961; Scharner and Zammit, 2011). Satellite cells provide new myonuclei during postnatal growth, and then become mitotically quiescent in mature muscle, while also retaining the ability to supply myoblasts for muscle hypertrophy and repair when called upon to do so (Yablonka-Reuveni, 2011).

In adult muscle, MuSCs can be identified by the transcription factor Pax7, which is expressed in all quiescent and proliferating satellite cells, across many different species (Yin et al., 2013). Prenatal and postnatal myogenesis are regulated by a family of basic helix-loop-helix transcription factors known as myogenic regulatory factors (MRFs). These include Myf5, MyoD, Myogenin and MRF4, and they orchestrate the progression of satellite cells through the myogenic program, along with Pax7 (Yablonka-Reuveni et al., 2008; Schmidt et al., 2019; Shirakawa et al., 2022). While the dynamics of their temporal and spatial expression have allowed for the characterization of a sequential and hierarchical relationship between these factors, the full scope of their mechanisms of action is still being worked out. In general, upregulation of Myf5 marks the earliest phase of myogenic commitment, followed by expression of MyoD, which marks a majority of activated and proliferating satellite cells. Downregulation of Pax7 and concomitant upregulation of MRF4 and myogenin then signal the terminal differentiation of satellite cells into skeletal muscle progenitor cells (Relaix and Zammit, 2012; Wang and Rudnicki, 2012; Forcina et al., 2020; Relaix et al., 2021).

## Satellite cell differentiation and self-renewal

**FIGURE 2:**
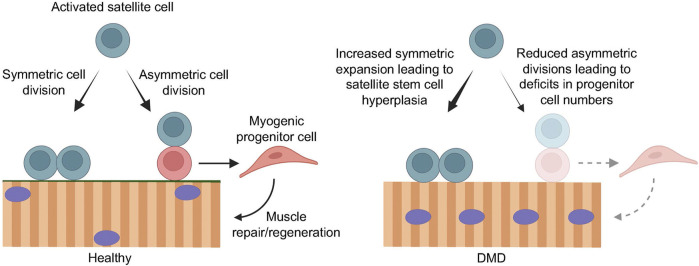
Loss of dystrophin compromises efficient muscle regeneration. In healthy muscle (left), regenerative myogenesis is initiated by activated satellite cells that undergo symmetric cell division to maintain the muscle stem cell pool, as well as asymmetric cell division to give rise to myogenic progenitor cells that expand and contribute to efficient muscle repair and regeneration. In the context of DMD (right), the loss of dystrophin in activated satellite cells results in impaired cell polarity cues, leading to mitotic defects and reduced asymmetric cell divisions. Consequently, the balance favors symmetric stem cell expansion, resulting in satellite cell hyperplasia, and greatly reduced asymmetric cell divisions and myogenic progenitor cell numbers. This impairs muscle regeneration, leading to progressive muscle degeneration and wasting. Created with BioRender.com.

While satellite cell differentiation is the process by which newly formed myofibers are available for muscle repair and regeneration, satellite cell self-renewal is required to replenish the stem cell pool. Maintenance of this balance between differentiation and self-renewal is critical for muscle homeostasis. Following activation, a satellite cell can undergo either symmetric or asymmetric cell division (Figure 2). In symmetric cell division, one stem cell can generate two functionally identical daughter cells. In asymmetric satellite cell division, the asymmetric segregation of intrinsic cell fate determinants prior to cell division can give rise two daughter cells with divergent cell fates, where one cell will be a self-renewing stem cell, while the other will be a committed myogenic progenitor that can differentiate and fuse, either with each other to form new myotubes or to existing muscle fibers (Kuang et al., 2007; Dumont et al., 2015a; Feige et al., 2018) (Figure 2). Consequently, a defect in self-renewal ability results in depleted satellite cell numbers and the stem cell pool, and impaired muscle regeneration capacity. Reduced asymmetric divisions, on the other hand, lead to a reduced rate of myogenic progenitor cell generation, which also impairs muscle regeneration (Figure 2). As such, a tightly regulated balance between satellite cell self-renewal and differentiation is paramount for maintaining the stem cell pool and for generating enough progenitors to support efficient growth and regeneration of muscle.

## Satellite cells in DMD

Our previous work established that, in addition to the myofiber, dystrophin protein is also expressed at high levels in activated satellite cells where it regulates satellite cell fate and myogenesis (Dumont et al., 2015b). Dystrophin binds the cell polarity effector Mark2 (also known as Par1b) in activated satellite cells. This interaction promotes the asymmetric segregation of the cell polarity regulator Pard3 to the opposite pole of the cell, and establishes the polarized distribution of the PAR complex (Dumont et al., 2015b). This results in the apicobasal orientation of mitotic spindle and asymmetric cell division. Asymmetric cell division enables satellite cells to maintain their reserve cell pool (via the self-renewing stem cell), and contribute to the myogenic progenitor population at the same time (via the differentiating cell).

In the absence of dystrophin in DMD satellite cells, cellular polarity is disrupted, resulting in increased mitotic stress, and abnormal mitotic spindle orientation and cell division. Further, there is a loss of asymmetric cell divisions and a reduction in myogenic progenitor cell numbers and muscle regeneration (Dumont et al., 2015b; Chang et al., 2016; Feige et al., 2018).

## Pathophysiology of the satellite cell phenotype in DMD

**FIGURE 3:**
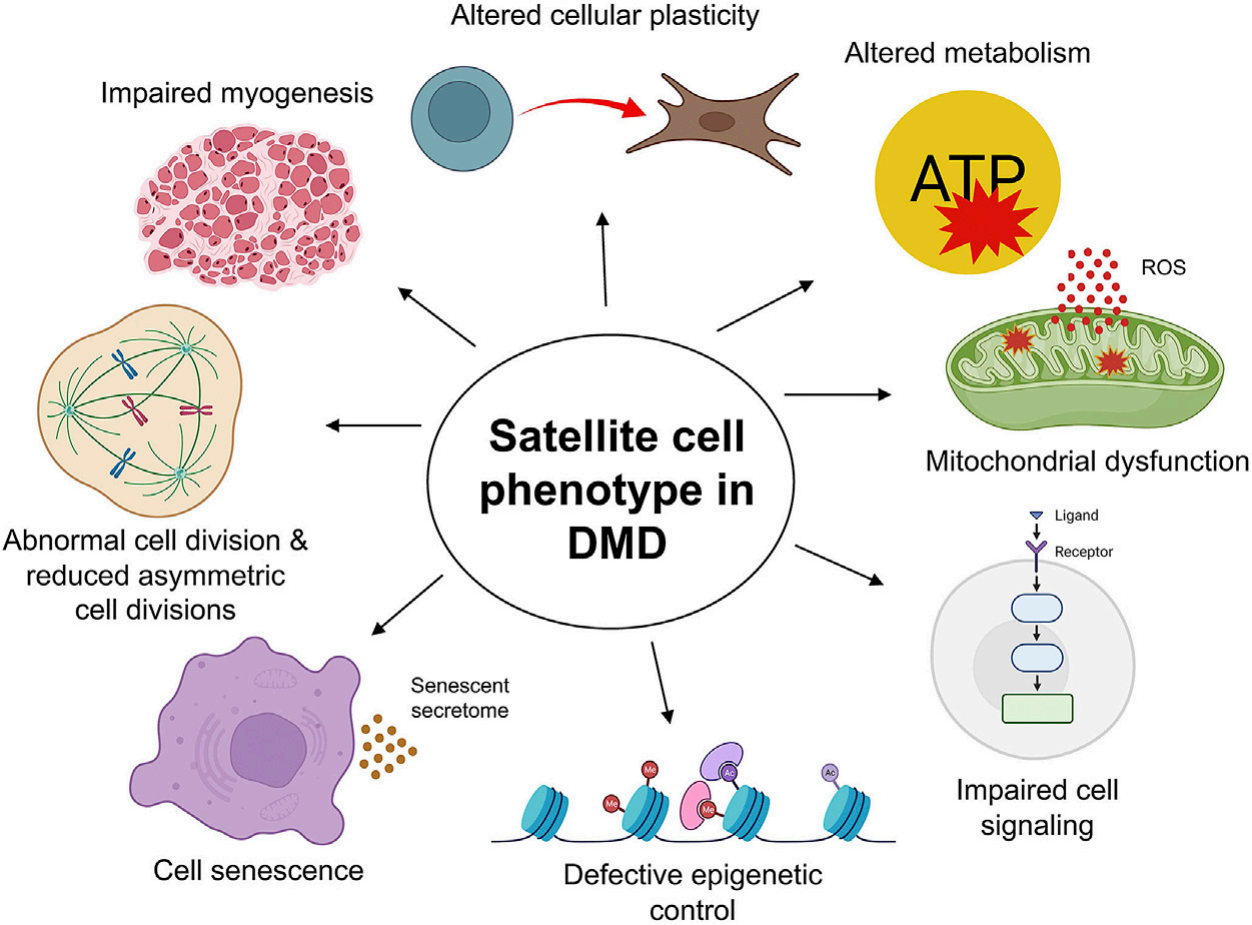
The satellite cell phenotype in Duchenne muscular dystrophy (DMD). The cell-intrinsic phenotype of satellite cells in DMD muscle manifests in a variety of ways. The loss of dystrophin impairs satellite cell division, leading to reduced asymmetric cell divisions, myogenic progenitor cells, and compromised regenerative myogenesis. Altered cellular plasticity of satellite cells in response to chronic injury and inflammation contributes to connective tissue deposition. Aberrant mitochondrial morphology, function, and metabolic profile lead to reduced proliferation and functional loss of satellite cells, further exacerbating DMD pathology, particularly in advanced stages of the disease. Elevated expression of senescence markers, mitotic defects, and increased oxidative stress and inflammation are all characteristic of DMD satellite cells and muscle pathology. Further, satellite cells maintain tightly regulated epigenetic control and cell signaling for optimal function. Loss of dystrophin in DMD satellite cells compromises these regulatory mechanisms, affecting satellite cell function at all stages, including maintenance of quiescence, activation, division, proliferation, and differentiation, ultimately contributing to the severe disease pathology in DMD. Created with BioRender.com.

It is now well established that muscle fibers from human DMD patients as well as dystrophin-deficient mdx mice show significantly elevated numbers of Pax7^+^ satellite cells (Kottlors and Kirschner, 2010; Bankole et al., 2013; Dumont et al., 2015b; Ribeiro et al., 2019) (Figure 2). This is contrary to the initial hypothesis that satellite cell numbers are exhausted in DMD (Heslop et al., 2000; Luz et al., 2002). Instead, accumulating evidence from our group and others points to a cell intrinsic defect in dystrophic satellite cells, resulting in reduced asymmetric cell division and myogenic commitment, as well as increased mitotic stress, abnormal cell division events and cellular senescence (Figure 3).

## Reduced asymmetric cell division and impaired myogenesis

In dystrophic muscle, the expression of Mark2 is reduced, and the PAR complex remains uniformly distributed, which greatly affects cell polarity establishment and asymmetric cell division. We have previously shown that satellite cells from mdx mice display a 5-fold reduction in the proportion of asymmetric divisions (Dumont et al., 2015b). Consequently, the number of myogenic progenitors that are available for efficient muscle regeneration is also significantly reduced (Figure 2). Further exacerbating this defect is the altered differentiation kinetics of dystrophic myoblasts. Yablonka-Reuveni and Anderson previously showed that dystrophic myoblasts in cell culture displayed an earlier reduction in MyoD expression and an earlier than normal expression of myogenin, compared to wild-type cells (Yablonka-Reuveni and Anderson, 2006). Dystrophic cells also differentiated more rapidly in cell culture than did wild-type cells. Consistent with these early findings, more recent evidence using transcriptomic, genome-scale metabolic modeling and functional analyses have also established cell-autonomous defects in DMD myoblasts. Gosselin et al. showed significantly altered expression of 170 genes in mdx myoblasts, including MyoD and key genes controlled by MyoD such as myogenin, Mymk (myomaker), Mymx (myomixer), epigenetic regulators, extracellular matrix interactors, calcium signaling and fibrosis genes (Gosselin et al., 2022). Functionally, these changes resulted in increased myoblast proliferation, altered chemotaxis, and accelerated differentiation in myoblasts derived from mdx mice as well as human DMD patients (Gosselin et al., 2022). Myoblasts are the effector cells of muscle growth and repair, and these data underline the cell autonomous abnormalities in DMD that negatively impact cell cycle exit, migration to the site of muscle damage, and cell fusion; three processes that are critical for efficient myogenesis.

Myotubes derived from DMD myoblasts have also been observed to have reduced expression of sarcomeric genes, likely contributing to the characteristic sarcomeric instability seen in DMD skeletal muscle (Barthélémy et al., 2022). Moreover, DMD patient derived human induced pluripotent stem cells differentiated into myotubes have also been shown to undergo pronounced calcium ion (Ca^2+^) influx upon *in vitro* contraction (Shoji et al., 2015; Yoshioka et al., 2021). Increased susceptibility to sarcolemmal injury and excess calcium ion influx are generally regarded as the initial triggers for skeletal muscle damage in DMD, and these lines of evidence once again highlight the stem cell phenotype that contributes to DMD pathology.

## Mitochondrial dysfunction

Currently, there is a large body of data implicating mitochondrial dysfunction as a secondary contributor to muscle pathology in DMD (Vila et al., 2017; Ramos et al., 2020; Reid and Alexander, 2021; Bellissimo et al., 2022) (Figure 3). Moore et al. showed aberrant mitochondrial morphology, reduced cristae number, and large mitochondrial vacuoles in mdx mouse muscle prior to onset of myofiber necrosis, which progresses with disease severity (Moore et al., 2020). Further, microtubule disorganization has been noted in dystrophin-deficient mouse muscle which was associated with impaired ADP control of mitochondrial bioenergetics (Ramos et al., 2020). While a low-level production of reactive oxygen species (ROS) is required for cell-cell signaling in normal cells, dysfunctional mitochondria in dystrophic muscle release abnormal levels of ROS, which can then activate pro-inflammatory pathways which further exacerbate disease pathology (Chen et al., 2022b). Further, it has been shown using nuclear magnetic resonance spectroscopy, that such elevated ROS production and damaged mitochondrial membrane in human and mouse dystrophic mitochondria are associated with reduced ATP generation and oxidative capacity (Kuznetsov et al., 1998; Griffin et al., 2001; Sharma et al., 2003). And restoring mitochondrial bioenergetic function ameliorated the DMD pathology in mouse models (Pauly et al., 2012; Luan et al., 2021).

So what role do satellite cells play in this context? Onopiuk et al. (2009) previously showed that mdx myoblasts displayed reduced oxygen consumption and mitochondrial membrane potential, and elevated ROS formation. Matre et al. (2019) provided further insight into the role of dystrophin deficiency in satellite cells with respect to bioenergetics and stress resistance. By restoring dystrophin expression in mdx satellite cells via CRISPR/Cas9, they showed significantly reduced oxidative stress, ROS formation, and mitochondrial membrane potential in edited cells. Dystrophin restoration also improved mitochondrial function in mdx satellite cells in terms of oxygen consumption rate, ATP production, maximal respiratory capacity, and mitochondrial coupling efficiency. These dystrophin-restored mdx satellite cells also displayed increased tricarboxylic acid (TCA) cycle activity and levels of Krebs cycle metabolites such as citrate and malate. Finally, transplantation of these edited mdx satellite cells in dystrophic mice resulted in significantly better survival and cell engraftment compared to unedited mdx satellite cells (Matre et al., 2019).

In regenerating muscle, mitochondrial homeostasis is maintained by removing damaged mitochondria by mitophagy, and adding newly synthesized mitochondria derived from satellite cells to reconstitute the mitochondrial network in regenerating myofibers. Using a satellite cell transplantation model, Mohiuddin et al. (2020) show that dystrophic satellite cells have altered mitochondrial gene expression and content, and a significant reduction in mitochondrial respiration. Further, DMD satellite cells, which carry defective mitochondria, fuse with existing fibers to propagate their mitochondrial dysfunction during muscle regeneration (Pant et al., 2015; Mohiuddin et al., 2020). They also show that functional satellite cells from healthy donors can reconstitute the mitochondrial network in dystrophic host muscle and improve mitochondrial function. Therefore, the quality of the mitochondrial network of the satellite cells that are fusing to existing fibers during muscle repair determine the oxidative capacity of the muscle undergoing repair.

## Satellite cell senescence

Cell senescence is a permanent (i.e., irreversible) cell cycle arrest, which is a normal occurrence during embryonic development, tissue repair, and regeneration (Kumari and Jat, 2021). Once the growth and regeneration processes are completed, these senescent cells are eliminated. Cell senescence can be induced by stressors such as DNA damage stress, oxidative stress, etc. However, a progressive accumulation of senescent cells is a hallmark of muscle pathology, such as in DMD, due to elevated levels of oxidative stress and inflammation (Sugihara et al., 2020; Saito and Chikenji, 2021; Young et al., 2021). Senescent cells are metabolically active and secrete inflammatory cytokines and chemokines that lead to a further increase in chronic inflammation (Kwon et al., 2019). They can also induce other nearby non-senescent cells to undergo senescence by releasing juxtacrine and paracrine effectors, a phenomenon termed secondary senescence (Kirschner et al., 2020). Therefore, senescent cells can have widespread effects in exacerbating tissue pathologies.

In a recent study, Sugihara et al. (2020) provided new insights into the role of satellite cell senescence in DMD progression (Figure 3). They show elevated expression of senescence markers such as CDKN2A, p16, and p19 in a rat model of DMD, as well as skeletal muscle tissue of human DMD patients. By reversing senescence via genetic ablation of p16, the authors observed a functional recovery of satellite cells, enhanced muscle regeneration, and the amelioration of the DMD phenotype in the rat model (Sugihara et al., 2020). In addition, depleting senescent cells via pharmacological means in late-stage DMD rats resulted in significantly decreased expression of senescence markers and improved muscle regeneration. These results are evidence that the presence of senescent cells is detrimental and exacerbates disease progression in DMD.

In another study, mdx mice carrying shortened telomeres in satellite cells displayed severe muscular dystrophy that worsened with age (Sacco et al., 2010). In these mice, the cell-autonomous defect of satellite cells to maintain the damage-repair cycle of dystrophic muscle was further worsened by a decreased telomere length. Since telomere shortening is one of the causes of cell senescence, these data could be supportive of the deleterious impact of senescent cells in dystrophic muscle.

## Altered cellular plasticity

As mentioned previously, one of the hallmarks of DMD pathology is the excessive and dysregulated deposition of fibrotic tissue in skeletal muscle in response to chronic tissue injury and inflammation. The extent of the accumulation of connective and fat tissue often corresponds to the severity of the disease. TGFβ activity and the expression of its downstream signaling mediators (such as activated SMAD2/3) increase with age in mdx mice, contributing to impaired muscle regeneration and function (Vidal et al., 2008; Mann et al., 2011; Kharraz et al., 2014). While the role of muscle resident fibro-adipogenic progenitors (FAPs) that differentiate into fibroblasts and adipocytes and contribute to fibrosis has been well characterized, accumulating evidence of altered cellular plasticity of satellite cells shows that a fraction of these muscle stem cells can undergo a mesenchymal-like/fibrogenic transition in DMD muscle (Figure 3).

Previous *in vitro* data using satellite cells isolated from old (18-mo) mdx mice or from muscles of DMD patients showed that a fraction of these myoblasts was reprogrammed to attain a profibrotic function, producing higher amounts of collagen in culture compared to control cells (Ionasescu and Ionasescu, 1982; Alexakis et al., 2007). Subsequently, Biressi et al. (2014) provided *in vivo* evidence that satellite cells obtained from dystrophic muscles of mdx mice showed compromised myogenic potential and enhanced expression of fibrogenic genes due to increased TGFβ activity. They showed elevated canonical Wnt signaling in mdx mouse muscles which in turn induced the expression of TGFβ2. This Wnt-TGFβ2 signaling axis promoted the acquisition of fibrogenic features by myoblasts. Furthermore, *in vivo* inhibition of the TGFβ pathway in mdx mice with anti-TGFβ antibody or by drug treatment reduced the expression of fibrotic genes and markers such as collagen type I and fibronectin 1, and rescued the satellite cell phenotype and partially ameliorated the dystrophic pathology (Biressi et al., 2014).

Consistent with these data, Pessina et al. (2015) also provided evidence of TGFβ-induced alterations in cellular plasticity in advanced dystrophic muscles of humans and mice where a fraction of myogenic cells (in addition to endothelial and inflammatory cells) lost their cell identity and acquired the capacity to produce extra cellular matrix proteins such as α-smooth muscle actin, collagen type I, and fibronectin. Interestingly, this fibrogenic process in response to elevated TGFβ signaling also resulted in the acquisition of a mesenchymal progenitor multipotent status, where myogenic cells that had acquired fibrogenic traits were also more prone to undergo osteogenic or adipogenic conversions (Pessina et al., 2015). These alterations in cellular plasticity of dystrophic satellite cells directly contributed to impaired muscle regeneration and worsened disease pathology.

## Defective epigenetic control of satellite cell function

Satellite cells are subjected to strict epigenetic control at various stages of myogenesis (Segalés et al., 2015; Massenet et al., 2021; Cicciarello et al., 2022). For example, we now know that satellite cell quiescence is an active and reversible state, controlled by specific epigenetic mechanisms (Gopinath et al., 2014; Boonsanay et al., 2016; Li and Dilworth, 2016; García-Prat et al., 2020). Our group and others have shown that quiescent satellite cell activation, division, and proliferation are also under tight epigenetic control (Tapscott, 2005; McKinnell et al., 2008; Dilworth and Blais, 2011; Diao et al., 2012). Pax7 plays a critical role in the transcriptional regulation of satellite cells (Soleimani et al., 2012; Lilja et al., 2017). Moreover, Pax7 also triggers Myf5 synthesis in committed progenitor cells, via its interaction with the arginine methyltransferase CARM1 (coactivator-associated arginine methyltransferase 1). This interaction is crucial for establishing polarity-regulated gene expression during asymmetric satellite cell division by methylating Pax7 at several arginine residues, thereby leading to the expression of Myf5 (Kawabe et al., 2012; Saber and Rudnicki, 2022).

In a previous study, we showed that CARM1 is a specific substrate of p38γ, and that phosphorylation of CARM1 prevented its nuclear translocation (Chang et al., 2018; Saber and Rudnicki, 2022). β1-syntrophin, a component of the dystrophin-associated protein complex, is required for the basal localization of the p38γ/p-CARM1 complex in satellite cells. Specifically, p38γ localization is restricted to the basal surface by β1-syntrophin during asymmetric satellite cell division. This leads to the phosphorylation of CARM1, and subsequent inhibition of Myf5 activation (Chang et al., 2018).

Importantly, we also showed that in dystrophin-deficient mdx satellite cells, the p38γ and β1-syntrophin interaction was completely absent. Consequently, there was increased phosphorylation of CARM1, and decreased nuclear translocation and interaction with Pax7, leading to impaired epigenetic activation of Myf5 and reduced myogenic progenitor cell production (Chang et al., 2018).

In another study, Acharyya et al. (2010) showed an additional role for TNFα and NFκB to impact myogenesis by negatively regulating satellite cell activation through epigenetic silencing of notch 1 via hypermethylation of its promoter region. Moreover, mdx skeletal muscles show higher histone deacetylase (HDAC) activity compared to wild type mouse muscle (Renzini et al., 2022). And mdx satellite cells derived from single myofibers also show increased global deacetylase activity and higher expression levels of HDAC2 (Colussi et al., 2008). Furthermore, treatment with histone deacetylase inhibitors ameliorated dystrophic pathology (Bajanca and Vandel, 2017). Interestingly, it has been reported that nitric oxide (NO) donors also improved the dystrophic phenotype in a manner similar to histone deacetylase inhibitors, perhaps suggesting a common mechanism of action (Colussi et al., 2008). The absence of dystrophin in DMD muscle results in the loss of sarcolemmal localization and reduced expression of neuronal nitric oxide synthase (nNOS), a key component of the dystrophin associated protein complex that is involved in several functional roles in skeletal muscle including contraction, glucose uptake, blood flow regulation, and muscle regeneration (Lai et al., 2009). This loss of nNOS and subsequent nitric oxide signaling results in a decrease in NO-dependent S-nitrosylation of HDAC2 in mdx muscle. And restoring this NO-signaling-dependent inhibition of HDAC2 showed significant therapeutic effect in dystrophic mouse muscle (Colussi et al., 2008; Colussi et al., 2009).

## Restoring satellite cell function as a treatment strategy for DMD

Antisense oligonucleotide (AON) mediated exon skipping, Adeno associated virus (AAV) mediated gene therapy, gene editing using the CRISPR/Cas9 system, as well as cell therapy with dystrophin competent cells have all received considerable research focus in recent decades as viable treatment options for DMD (Sun et al., 2020; Duan et al., 2021; Happi Mbakam et al., 2022). However, these therapeutic strategies largely target the dystrophic myofibers, and do not address the cell-autonomous defects of dystrophin-deficient satellite cells that we have outlined thus far.

One challenge of CRISPR/Cas9 mediated gene editing in correcting the genetic defect of DMD cells is the long-term maintenance of the edited gene (Chen et al., 2022a). Satellite cell transduction and editing by CRISPR/Cas9 have traditionally been limited, and consequently, the edited nuclei may be diluted out as new muscle cells are generated. However, recent reports have explored the possibility of successfully editing satellite cells with the use of more efficient genetic tools. Kwon et al. (2020) reported that by systematically assessing a panel of AAV serotypes with different tissue tropisms, they identified AAV9 as having the highest transduction rate in satellite cells in mdx mice via both local injections and systemic tail-vein injections. Paired with muscle specific promoters, they were able to achieve up to ∼60% Cre-mediated recombination. Unfortunately, however, the percentage of total gene editing in satellite cells was drastically lower at ∼1.5% (Kwon et al., 2020). Consistent with these data, Nance et al. (2019) also reported using AAV9 to successfully perform gene editing and restore dystrophin expression in mdx satellite cells. In another recent study, Domenig et al. (2022) reported the use of a novel *in vitro* cellular model in combination with CRISPR/Cas9 gene editing to restore dystrophin expression in dystrophic mice. Specifically, they reprogrammed fibroblasts obtained from dystrophic mice into highly proliferative induced myogenic progenitor cells (iMPCs), and then performed CRISPR/Cas9 mediated exon skipping to correct the genetic mutation in these cells and restore dystrophin expression. Engraftment of these corrected DMD iMPCs into the muscle of dystrophic mice restored dystrophin expression *in vivo* and contributed to the muscle stem cell pool as well (Domenig et al., 2022).

There is accumulating evidence now showing that restoring satellite cell function can have tremendous therapeutic potential for DMD. One such strategy is to augment asymmetric satellite cell divisions and significantly increase the number of myogenic progenitors available for muscle regeneration. For example, the epidermal growth factor receptor (EGFR)—aurora kinase A (Aurka) signaling pathway is an alternative effector pathway to regulate asymmetric satellite stem cell divisions (Wang et al., 2019). Specifically, EGF treatment drives its receptor (EGFR) that is localized at the basal surface of satellite cells to recruit the mitotic spindle assembly protein Aurka and induce asymmetric divisions (Wang et al., 2019). Crucially, this cell polarity pathway is independent of dystrophin or the DAPC, and therefore, can rescue the deficit in asymmetric stem cell divisions seen in DMD satellite cells. Indeed, mdx mice treated with exogenous EGF showed significantly increased progenitor numbers, enhanced muscle regeneration as well as muscle strength (Wang et al., 2019). Lending further credence to this notion, Flanigan et al. (2021) have recently identified PARD6G as a genetic modifier capable of influencing disease severity in DMD patients. In a large genome-wide association study (GWAS) of loss of ambulation in patients, the group discovered PARD6G, a member of the PAR polarity complex (PAR3-PAR6-aPKC) that involved in the control of asymmetric cell division of satellite cells, as a genetic modifier in a substantial cohort of DMD patients who showed significantly prolonged ambulation (Flanigan et al., 2021).

Various other signaling pathways involved in cell polarity establishment and satellite cell proliferation and differentiation could also be targeted as a therapeutic strategy. For example, the canonical notch signaling pathway governs satellite cell fate decisions, cell proliferation and induction of differentiation (Gioftsidi et al., 2022). Further, interactions between a variety of notch ligands and receptors need to be precisely regulated temporally and spatially for optimal results. Several lines of evidence show that impaired notch signaling contributes to the pathogenic mechanisms of DMD (Church et al., 2014; Jiang et al., 2014; Den Hartog and Asakura, 2022). Mu et al. (2015) showed an overactivation of notch signaling in dystrophic mouse skeletal muscle, and the repression of notch signaling reduced the depletion and senescence of muscle progenitor cells and restored myogenic capacity. Moreover, it was revealed that overexpression of Jagged 1, a notch ligand, acted as a genetic modifier that dramatically improved muscle regeneration and function in two dystrophic “escaper” dogs that were phenotypically normal (Vieira et al., 2015). The notch signaling pathway is also involved in asymmetric satellite cell divisions via its interacting partner numb, which is asymmetrically inherited by the committed daughter cell (Conboy and Rando, 2002). It remains to be seen whether pharmacological modulation of this notch/numb signaling dynamic can rescue the asymmetric cell division deficit observed in DMD satellite cells.

The Jak/Stat signaling pathway is another example of a signaling axis that regulates asymmetric cell divisions and drives myogenic progenitor formation (Price et al., 2014). Stat3 signaling promotes myogenic lineage progression via its regulation of MyoD (Tierney et al., 2014). And the use of Stat3 inhibitors has been shown to promote tissue repair and partially restore muscle regeneration in dystrophic muscle (Sala and Sacco, 2016). We have also previously shown that Wnt7a, which is known to stimulate the planar cell polarity pathway via its interaction with the Fzd7 receptor, can also be manipulated to promote satellite stem cell expansion and improve muscle regeneration and function in mdx muscle (Le Grand et al., 2009; von Maltzahn et al., 2012).

As discussed in the previous section, the confluence of chronic inflammation, oxidative stress, and satellite cell senescence greatly exacerbates the disease pathology in DMD. The senescence associated secretory phenotype (SASP) is where senescent cells secrete various cytokines such as TGFβ and Interluekin-6 (IL-6) that contribute to fibrosis and inflammation. Since SASP of senescent muscle satellite cells compromises muscle regeneration, removal of these senescent cells is expected to be of therapeutic value in DMD muscle. To test this hypothesis, Sugihara et al. (2020) treated 8-mo old DMD rats with the senolytic drug ABT263, which specifically depletes senescent cells. ABT263 treatment successfully decreased the expression of senescence markers and senescent cells, and increased the number of newly regenerating muscle fibers in treated dystrophic muscle (Sugihara et al., 2020). In another recent study, Moiseeva et al. (2023) developed a lifetime atlas of *in vivo* senescent cells in regenerating skeletal muscle, using single-cell transcriptomics and a method to separate senescent cells from subsets of various niche cell types. They show increased presence of senescent cells in the dystrophic muscles of mdx mice, and reducing the accumulation of senescent cells in mdx mice by treating with the senolytic compounds dasatinib and quercetin increased the size of regenerating myofibers and decreased inflammation and matrix deposition (Moiseeva et al., 2023). Furthermore, they show that senescent cells can reduce satellite cell expansion through paracrine pro-inflammatory and pro-fibrotic SASP activity, which may impair muscle regeneration, particularly in advanced stages of the disease (Moiseeva et al., 2023).

Increased levels of Ca^2+^ influx lead to myofiber necrosis and a severe inflammatory response in DMD. This response is mainly mediated by the NF-kB pro-inflammatory pathway. TNF-α and IL-6 are primarily responsible for regulating this pathway. While current strategies that target muscle inflammation in DMD include the use of glucocorticoids such as prednisone, prednisolone, and deflazacort, their long-term usage carries severe immune modulatory side effects (Szabo et al., 2021). Several small molecule drug candidates are currently in clinical trials that aim to modulate these pro-inflammatory immune responses in DMD muscle, and improve the satellite cell niche and promote muscle regeneration. These also include histone deacetylases inhibitors, that have been shown to be especially effective at treating inflammation in dystrophic muscle over corticosteroids. In several preclinical studies, small molecule HDAC inhibitors yielded significant functional and morphological benefits to dystrophic muscle, including decreasing the inflammatory infiltrate, and increasing muscle size, quality, and function, as well as improving the therapeutic efficacy of gene therapy approaches (Consalvi et al., 2011; Sincennes et al., 2016; Licandro et al., 2021; Bizot et al., 2022). Importantly, they have also been shown to directly modulate the function of satellite cells. For example, Murray et al. investigated the role of dietary tributyrin, an HDAC inhibitor, on satellite cell activity and muscle regeneration in a piglet model. They showed that tributyrin treatment in these piglets enhanced the terminal differentiation of satellite cells and improved muscle growth (Murray et al., 2018; Murray et al., 2021). Moreover, treatment with trichostatin A (TSA), valproic acid, or sodium butyrate, which are all inhibitors of class I and II HDACs, has been shown to enhance myogenic differentiation and improve the efficiency of myoblast recruitment and fusion (Iezzi et al., 2002; Iezzi et al., 2004).

## Concluding remarks

It is now evident that a truly effective treatment for DMD will need to account for the myofiber phenotype as well as the muscle stem cell phenotype. In the absence of dystrophin, the myofiber phenotype manifests in the form of contraction-induced sarcolemmal injury, membrane leakage, impaired muscle contraction and loss of force, increased Ca^2+^ influx, inflammation, progressive muscle degeneration, and fibrotic tissue deposition. The muscle stem cell phenotype, on the other hand, leads to reduced asymmetric stem cell divisions and impaired myogenesis, accelerated differentiation, altered metabolism and mitochondrial dysfunction, satellite cell senescence, defective epigenetic control, and impaired cell signaling (Figure 3). These phenotypes converge to result in a devastating and fatal muscle disease that currently has no cure. While groundbreaking research into gene therapy (including gene replacement and gene editing) aim to tackle the primary defect of dystrophin loss in myofibers in DMD, these strategies fail to adequately address the muscle stem cell phenotype. Here we have outlined several exciting and viable treatment avenues to target the stem cell phenotype and to ameliorate the dystrophic pathology and improve the quality of life for patients.

However, it is worth noting that a disease with such widespread pathogenesis will require a synergistic therapeutic approach for optimal results. For example, improving the stem cell phenotype by increasing asymmetric cell divisions and the number of myogenic progenitor cells available for muscle regeneration will need to be executed in a way that satellite cell self-renewal and replenishment of the stem cell pool are not compromised. Otherwise, the stem cell pool may be exhausted, resulting in impaired muscle regeneration, once again. Further, as we have described here, dystrophic satellite cells carry an inherent pathology in terms of their altered gene expression, metabolism, elevated inflammation and oxidative stress, and propensity for cell senescence. While increasing myogenic progenitor numbers in this dystrophic milieu will undoubtedly improve myogenesis and muscle function, these progenitors will continue to propagate their intrinsic defects via fusion to each other or existing myofibers. However, it remains to be determined whether a pharmacological rescue of the impairment in asymmetric division will ameliorate these intrinsic deficits. Therefore, we must continue our efforts to lay the groundwork for a more comprehensive understanding of the pathophysiology of this disease, while leaving no stone unturned in our progress towards an all-encompassing treatment for these patients.
